# Species‐ and Level‐Dependent Modulation of Growth and Skin and Intestinal Mucosal Microbiota in Rainbow Trout Fed Mushroom Stem Meals

**DOI:** 10.1155/anu/9264855

**Published:** 2026-05-28

**Authors:** Samira Reinoso, Carl John Saromines, Silvia Torrecillas, Maria Luisa Tello Martín, Margarita Pérez Clavijo, Enric Gisbert

**Affiliations:** ^1^ IRTA, Aquaculture Program, Centre de La Rápita, La Rápita, 43540, Catalonia, Spain, irta.cat; ^2^ Mushroom Technological Research Center of La Rioja (CTICH), La Rioja, Spain

**Keywords:** *Agaricus bisporus*, circular ingredients, microbiota, *Pleurotus ostreatus*, skin mucus, sustainable ingredients, zero waste

## Abstract

Mucosal microbiomes play key roles in fish physiology by supporting digestion, nutrient absorption, metabolism, immune modulation, and pathogen defense. This study evaluated the effects of mushroom stem meals from *Agaricus bisporus* (AB) and *Pleurotus ostreatus* (PO) as nutritional alternatives to soybean meal (SBM) on growth performance and mucosa‐associated microbiota in rainbow trout (*Oncorhynchus mykiss*). A 60‐day feeding trial was conducted with juveniles (22.1 ± 0.3 g) at 16.02 ± 0.19°C using isoproteic (47%) and isolipidic (23%) diets containing 50 or 100 g kg^−1^ AB or PO meals (AB5, AB10, PO5, and PO10), plus a control (CTRL) diet without mushroom meals. Microbial communities in feed, anterior and posterior intestine (PI), and skin mucus (SK) were analyzed by 16S rRNA (V3–V4) sequencing, and functional pathways were inferred. Growth responses were mushroom meal type‐ and level‐dependent. Fish fed the AB5 diet showed similar growth to the CTRL group (*p* > 0.05), while those fed AB10, PO5, and PO10 diets grew 8%, 25%, and 60% less, respectively (*p* ≤ 0.05). Feed intake (FI) was unaffected by AB meal but reduced by PO5 and PO10 diets (3% and 40%, respectively). AB diets maintained intestinal alpha diversity and community structure, whereas PO diets increased alpha diversity and altered beta diversity, favoring fermentative and opportunistic taxa while reducing beneficial fermenters. *Mycoplasma* abundance correlated positively with growth performance in both intestinal regions, while *Bradyrhizobium* abundance and Shannon diversity index correlated negatively in the anterior intestine (AI). Functional predictions showed enrichment of lipid and xenobiotic metabolism pathways in PO10‐fed fish, while AB10 enhanced carbohydrate‐related functions. SK microbiota diversity remained stable despite compositional shifts, with increases in fermentative taxa and reductions in potential pathogens. Overall, AB meal showed greater compatibility with rainbow trout growth performance, feed efficiency, and gut microbial homeostasis, whereas high PO inclusion impaired growth by reducing FI and inducing marked changes in the gut community structure. These findings suggest that AB meal at low inclusion levels may be used as an alternative to SBM without substantial effects on key performance indicators and gut microbiota in rainbow trout.

## 1. Introduction

Microbiota refers to the assemblage of microorganisms inhabiting a defined environment, where they fulfill essential ecological and functional roles [1]. In fish, microbial communities colonize internal mucosal surfaces, such as the gastrointestinal tract, as well as external surfaces, including the olfactory organ, gills, and skin. These surfaces also serve as primary entry points for pathogens [2]. These bacterial communities inhabiting host’s mucosae contribute to host physiology by supporting nutrient digestion, absorption, metabolism, immune modulation, and pathogen defense, thereby directly influencing fish condition, health, and welfare [3–5]. Moreover, recent studies suggest that microbiota may influence distant mucosal sites, such as the skin, through a gut–skin axis regulated by microbial metabolites (e.g., short‐chain fatty acids, SCFAs) and immune signaling [6]. In higher vertebrates, dysregulation of this axis has been linked to inflammatory skin disorders [7], and growing evidence suggests that a similar interplay may exist in fish [2, 8].

Because of its pivotal physiological functions, intestinal microbiota modulation has become a major focus in aquaculture nutrition and health. Among the wide range of intrinsic and extrinsic factors shaping these communities, the diet is the primary driver, exerting rapid and often region‐specific effects along the gastrointestinal tract [9–12]. Recent studies have shown that the replacement of conventional protein sources with novel ingredients can alter the intestinal microbiota and concomitantly modulate immune‐related gene expression and disease resistance in rainbow trout, such as when using black soldier fly meal as a dietary ingredient [13]. Moreover, dietary inputs can also affect the skin mucus (SK) microbiota [8, 14], and bacterial communities inhabiting the fish skin have also been proposed as non‐invasive biomarkers for assessing fish health and condition [15]. Although no direct or drastic effects on SK microbiota have been reported, certain dietary components may alter mucus composition [16], enhance disease resistance by regulating immune‐related gene expression and activity [17, 18], and increase the antibacterial activity of the SK [19, 20], either through microbial modulation [2, 21] or the presence of antimicrobial components [22]. These findings highlight the interconnection among mucosal tissues and the functional relevance of the gut–skin axis in host–microbiota interactions.

The aquafeed industry is shifting towards formulating nutritionally balanced, economically viable, and environmentally sustainable feeds less dependent fisheries; thus, the identification of alternative raw materials has become a key research priority [23–25]. In this context, the use of microbiome analyses, integrating gut and skin‐associated communities together with growth performance metrics, provides a holistic framework to evaluate the suitability of new feed ingredients beyond the conventional key performance indicators associated with growth and feed performance [26]. Among the potential alternative ingredients, agriculture by‐products, especially those from the mushroom industry, represent promising feed ingredients, as these materials are often discarded during commercial processing [27, 28]. Their regular availability and valorization as protein‐rich ingredients in aquafeed formulations offer a sustainable approach that supports waste reduction, circular bioeconomy principles, and nutritional adequacy in aquaculture feeds.

A recent study evaluated the digestibility of stem meals from two common mushroom species, the white button mushroom (*Agaricus bisporus* [AB]) and the oyster mushroom (*Pleurotus ostreatus* [PO]), using both in vitro and in vivo digestibility trials, revealing promising results in terms of ingredient and diet digestibility, as well as growth performance and feed efficiency [29]. Furthermore, the inclusion of the above‐mentioned mushroom stem meals (300 g kg^−1^) also promoted beneficial shifts in rainbow trout gut microbiome [30]. These results provided preliminary evidence that mushroom by‐products, biomass that is typically discarded during food processing, could serve dual roles as both sustainable protein sources and functional feed ingredients that support gut health. In this line, even though research on mushroom‐derived ingredients in aquafeeds is still limited, several studies indicate that their inclusion as dietary ingredients may beneficially modulate host microbiota, enhancing microbial diversity and promoting beneficial taxa such as lactic acid bacteria (LAB) [30–33]. These microbial changes have been linked to improved digestive efficiency, immune competence, and overall host’s health [9, 34, 35], suggesting that mushrooms may exert prebiotic‐like effects when used in basal feed formulations. Similarly, the incorporation of mushroom stem meals into fish diets has been associated with enhanced bactericidal activity in the SK [19], which may reflect microbiota‐mediated stimulation of host defense mechanisms. Together, these findings underscore the potential of mushroom by‐products as nutritionally functional feed ingredients capable of influencing both intestinal and skin‐associated microbiota, ultimately contributing to improved growth, health, and welfare in aquaculture species.

Therefore, to further evaluate their potential as alternative feed ingredients for aquafeed formulation based on the preliminary results of Saromines et al. [29], the present study aimed to determine the optimal inclusion levels of mushroom industry by‐products as a replacement for soybean meal (SBM), based on their effects on growth performance and skin and gut microbiota. The mushroom meals were derived from the edible by‐products of AB and PO. We hypothesized that these ingredients would exert level‐dependent effects on the growth performance and on the intestinal and SK microbiota of juvenile rainbow trout (*Oncorhynchus mykiss*).

## 2. Materials and Methods

### 2.1. Experimental Diets

Discarded mushroom stems from AB and PO used in the current study were provided by mushroom farms from La Rioja region (Spain) and processed by the Mushroom Technological Research Center of La Rioja (CTICH, Autol, Spain) as previously described by Saromines et al. [29]. The levels of dietary inclusion of AB and PO meals in isoproteic and isolipidic diets (crude protein [CP]: 47%, crude fat: 23%) were fixed at 50 and 100 g kg^−1^ based on previous results [29], and their inclusion was done at the expense of SBM (47.4% CP and 2.6% of lipids, Cargill, Spain), replacing 25% and 50% of SBM, which corresponded to the dietary inclusion of 50 and 100 g kg^−1^ of AB and PO meals. The experimental groups were as follows: CTRL (0 g kg^−1^ mushroom stem meal), AB5 (50 g kg^−1^ AB stem meal), AB10 (100 g kg^−1^ AB stem meal), PO5 (50 g kg^−1^ PO stem meal), and PO10 (100 g kg^−1^ PO stem meal). The experimental diets (pellet size: 4 mm) were formulated and produced by Sparos Lda. (Portugal). The composition of the mushroom stem meals is presented in Table 1, and the formulation and proximate composition of the experimental diets are shown in Table 2.

**Table 1:** Proximate composition, amino acid profile, and glucan content on a dry matter basis (DM) of mushroom stem meals from *Agaricus bisporus* and *Pleurotus ostreatus*.

Proximate composition, % DM	Mushroom stem meals	
	*Agaricus bisporus*	*Pleurotus ostreatus*
Dry matter	91.5	89.4
Crude protein	21.1	10.8
Crude lipids	3.8	3.0
SFA	1.0	0.5
MUFA	0.2	0.3
n‐6 PUFA	2.5	2.2
n‐3 PUFA	0.0	0.0
Ash	17.1	9.0
Gross energy, cal/g	3376	3525
Phosphorous, mg/g	6.2	3.3
Calcium, mg/g	34.7	0.7
Amino acids, % DM		
Aspartic acid	1.19	0.66
Threonine	0.59	0.34
Serine	0.55	0.34
Glutamic acid	2.28	1.54
Glycine	0.64	0.36
Alanine	1.55	0.69
Valine	0.58	0.31
Methionine	1.02	0.39
Isoleucine	0.45	0.24
Leucine	0.81	0.47
Tyrosine	0.38	0.22
Phenylalanine	0.60	0.32
Lysine	1.25	0.64
Histidine	0.35	0.20
Tryptophan	0.07	0.03
Cysteine	0.17	0.07
Arginine	3.65	1.10
Proline	0.80	0.27
Glucans composition^a^, %DM		
Total glucans	11.67	37.46
Alpha‐glucans	5.96	7.40
Beta‐glucans	5.72	30.06

^a^The glucan contents of mushroom stem meals were analyzed using a β‐glucan Assay Kit (K‐YBGL, Megazyme, Michigan, USA) according to the manufacturer’s protocol.

**Table 2:** Diet formulation and proximate composition of the experimental diets for rainbow trout (*Oncorhynchus mykiss*).

Ingredients, (%)	Experimental diets				
	CTRL	AB5	AB10	PO5	PO10
Fishmeal super prime^a^	10.00	10.00	10.00	10.00	10.00
Poultry meal^b^	8.00	8.00	8.00	8.00	8.00
Blood meal^c^	2.00	2.00	2.00	2.00	2.00
Soybean meal 48^d^	12.00	9.00	6.00	9.00	6.00
*Agaricus bisporus* meal^e^	—	5.00	10.00	—	—
*Pleurotus ostreatus* meal^e^	—	—	—	5.00	10.00
Soy protein concentrate^f^	14.00	14.00	14.00	14.00	14.00
Pea protein concentrate^g^	3.10	3.10	3.10	3.10	3.10
Corn gluten meal^h^	5.00	5.00	5.00	5.00	5.00
Wheat meal^i^	6.00	6.00	6.00	6.00	6.00
Wheat gluten^j^	10.00	10.60	11.30	10.70	11.60
Wheat starch^k^	7.00	4.50	1.90	4.40	1.60
Fish oil^l^	6.60	6.60	6.60	6.60	6.60
Rapeseed oil^m^	13.80	13.70	13.60	13.70	13.60
Vitamin and mineral premix^n^	1.00	1.00	1.00	1.00	1.00
Antioxidant^o^	0.18	0.18	0.18	0.18	0.18
Monoammonium phosphate^p^	1.00	1.00	1.00	1.00	1.00
L‐Lysine 99%^q^	0.20	0.20	0.20	0.20	0.20
DL‐Methionine^r^	0.10	0.10	0.10	0.10	0.10
Yttrium oxide^s^	0.02	0.02	0.02	0.02	0.02
Proximate composition, % DM					
Crude protein	47.71	47.10	47.18	47.50	46.90
Crude fat	24.03	23.29	23.51	24.89	23.28
Crude fiber	1.01	1.30	1.46	1.51	1.82
Ash	4.98	5.92	6.23	5.32	5.71
Gross energy, MJ kg^−1^ feed	23.40	23.38	23.52	23.32	23.76

^a^Fish meal Super Prime (Diamante): Pesquera Diamante, Peru; CP: 66.3%; CL: 11.5%.

^b^Poultry meal: SAVINOR UTS, Portugal; CP: 62.4%; CL: 12.5%.

^c^Blood meal: SONAC BV, The Netherlands; CP: 89.1%; CL: 0.4%.

^d^Soybean meal 48: CARGILL, Spain; CP: 47.4%; CL: 2.6%.

^e^Mushroom steam meals: CTICH, Autol, Spain; proximal composition in shown in Table 1.

^f^Soy protein concentrate (Soycomil P): ADM, The Netherlands; CP: 62.2%; CL: 0.7%.

^g^Pea protein concentrate (Lysamine GPS): Roquette, France; CP: 78.1%; CL: 8.3%.

^h^Corn gluten meal: COPAM, Portugal; CP: 61.2%; CL: 5.2%.

^i^Wheat meal: Molisur, Spain; CP: 11.7%; CL: 1.6%.

^j^Wheat gluten (VITAL): Roquette, France; CP: 80.4%; CL: 5.8%.

^k^Wheat starch (MERITENA 200): Tereos, France; CP: 0.4%; CL: 0.1%.

^l^Fish oil: Sopropêche, France.

^m^Rapeseed oil: JC Coimbra, Portugal.

^n^Vitamin and mineral premix (WISIUM MIX AQUA 1.5%): Premix Lda., Portugal.

^o^Antioxidant (VERDILOX): Kemin Europe NV, Belgium.

^p^Monoammonium phosphate: Phosphea, Serbia.

^q^L‐Lysine 99%: Dongxiao Biotechnology, China.

^r^DL‐Methionine (Rhodimet NP99): ADISSEO, France.

^s^Yttrium oxide (Amperit): Höganäs Germany GmbH, Germany.

### 2.2. Fish Husbandry and Feeding Trial

Juvenile rainbow trout were obtained from Truchas de Leiza SL (Leiza, Navarra, Spain). After 1‐week acclimation, 800 fish with a body weight (BW) of 22.1 ± 0.3 g (mean ± standard deviation) and standard length (SL) of 13.2 ± 0.1 cm were randomly distributed into 20 0.5 m^3^ tanks (stocking density = 40 fish per tank, 0.44 kg/m^3^) connected to an IRTAmar water recirculating system with mechanical, biological filtration and UV water treatment. Fish were fed twice daily (08:00 and 14:00) with diets over an experimental period of 60 days at a feeding rate of 2%–3% of tank biomass. Feeding was conducted by means of automatic feeders (ARVO‐TEC T Drum 2000; Huutokoski, Finland). Uneaten feed pellets were collected daily, dried overnight in an oven (120°C) and the amount of ingested feed was determined daily (feed intake, FI). This information was used for determining the amount of feed for the following day, since feed ration was adjusted to guarantee 10%–15% of uneaten pellets. This approach allowed us to confirm that fish were fed ad libitum. Water quality parameters were monitored daily to maintain within the optimal ranges: temperature 16.02 ± 0.19°C, pH 7.93 ± 0.12, dissolved oxygen 7.20 ± 0.18 ppm, and salinity 1.65 ± 0.05 ppt. Additionally, ammonium (NH_4_^+^), nitrite (NO_2_), and nitrate (NO_3_) levels were measured weekly and maintained at 0.49 ± 0.73 ppm, 0.30 ± 0.14 ppm, and 51.43 ± 14.60 ppm, respectively. This study was reported in accordance with ARRIVE guidelines [36].

### 2.3. Sampling

At the end of the trial, fish were subjected to a 48‐h fasting period to empty the digestive tract contents and allow sampling of the autochthonous intestinal microbiota. All fish per tank were hand‐netted, sedated with 50 mg L^−1^ tricaine methane sulfonate (MS‐222; Pharmaq Ltd., UK), and then measured for BW and SL to calculate growth performance indicators. For microbiota analysis, four host‐associated environments/matrices were collected: feed (D), SK, anterior intestine (AI), and posterior intestine (PI), as described by Ruiz et al. [37]. SK samples were collected from six fish per treatment using two sterile swabs per fish by gently swabbing both flanks, from the caudal and dorsal regions towards the operculum. Swabs (catalog number: MFS‐98000KQ, MEIDIKE GENE, China) were placed in sterile 2 mL tubes, immediately frozen, and stored at −80 °C until DNA extraction. For gut microbiome studies, 12 fish per treatment were randomly selected, euthanized with an overdose of MS‐222 (300 mg L^−1^), eviscerated, and the entire intestine was dissected, frozen immediately, and stored at −80 °C for further DNA extraction for microbiome studies. Additionally, samples of each experimental diet (*n* = 3 per diet) were collected and stored under the same above‐mentioned conditions.

### 2.4. Growth Performance

The following key performance indicators related to somatic growth, body condition, and feed efficiency were calculated according to the following formulae:

• Weight gain (WG, g) = BW _final_ − BW _initial_.
• Fulton’s condition factor (K) = 100 × (BW _final_/SL^3^_final_).
• Specific growth rate (SGR, %) = 100 × [(ln BW _final_ − ln BW _initial_)/ 60].
• Feed conversion ratio (FCR) = FI per tank (g)/ WG per tank (g).

### 2.5. Proximate Composition of Experimental Diets

The proximate composition of the experimental diets was determined following the methods of AOAC [38]. Dry matter (DM) was measured by drying samples at 105°C for 14 h (AOAC 925.09), and ash content was determined by incineration in a muffle furnace (Nabertherm, Germany) at 500°C for 5 h (AOAC 942.05). CP was quantified using the Dumas combustion method with a nitrogen analyzer (FP‐528, Leco, USA; AOAC 968.06), and crude lipid (CL) was determined according to the procedure of Folch et al. [39]. Crude fiber (CF) was analyzed using an Ankom fiber analyzer (Ankom, USA) based on filter bags technology (AOAC 962.09), and gross energy (GE) was measured with an adiabatic bomb calorimeter (IKA, C‐400, Janke & Kunkel KG., Staufen I. Br., Germany; DIN 51900 standard).

### 2.6. DNA Extraction and Amplification

Prior to DNA extraction, frozen intestines were thawed and processed under sterile conditions. The AI was defined as the *ca*. 3 cm section immediately following the last pyloric cecum, while the PI was defined as *ca*. 3 cm section anteriorly located from the anal opening. Each section was aseptically opened lengthwise, and *ca*. 200 mg of intestinal mucosa was scraped with a sterile scalpel and transferred into sterile 2 mL tubes for DNA extraction. The rest of the tissues were kept as back‐up samples.

DNA was extracted from four sample types: D, SK, AI, and PI. Approximately 200 mg of each sample were processed using the DNeasy PowerSoil Pro Kit (Ref. 47016, QIAGEN, Germany) according to the manufacturer’s instructions. DNA quality was assessed based on concentration (20–300 ng μL^−1^) and purity (A260/A280 ratio: 1.80–2.00) using a NanoDrop‐2000 spectrophotometer (Thermo Fisher Scientific, United States). The V3–V4 hypervariable regions of the 16S rRNA gene were amplified using primers 341F (5′‐CCTACGGGNGGCWGCAG‐3′) and 805R (5′‐GACTACHVGGGTATCTAATCC‐3′), employing Q5 High‐Fidelity DNA Polymerase (catalog number: M0491L, New England BioLabs, United States) as described by Ruiz et al. [40]. Amplicons were subsequently sequenced on an Illumina MiSeq platform (2 × 300 bp paired‐end). Raw sequencing data are available in the European Nucleotide Archive (ENA) under project accession number PRJEB102050.

### 2.7. Bioinformatic Analysis

Bioinformatic processing of the sequenced amplicons was performed as follows: raw FASTQ files were first trimmed to remove forward and reverse primers using QIIME2 (v2022.2) [41]. Subsequent analyses and visualizations were conducted in R (v4.2.2). Primer‐free reads underwent quality filtering and denoising with the DADA2 package (v1.32.0) [42], applying trimming parameters to retain only high‐quality sequences (*Q* score ≥ 30). Error rates were estimated from a subset of 1 × 10^8^ reads, followed by merging of paired‐end reads. Chimera removal and inference of amplicon sequence variants (ASVs) were then performed. Taxonomic classification was assigned against the SILVA reference database (v138.1) [43] with a minimum bootstrap confidence of 80%. Reads identified as mitochondrial or chloroplast sequences were removed from downstream analyses. Rarefaction was applied to the lowest read count observed across samples (28,534 reads), and consequently, four samples failing to meet such quality criteria were excluded from further analyses. A distance matrix was generated using the neighbor‐joining method, and a phylogenetic tree was constructed with the *phyloseq* package (v1.48.0) [44].

Venn diagram, alpha, and beta diversity analyses were performed using the *microeco* package (v1.9.1) [45]. Venn diagrams illustrated the number of shared ASVs among groups and their relative abundances (RA). Alpha diversity was quantified based on observed richness, Shannon, and Faith’s phylogenetic indexes. Beta diversity was calculated using both unweighted and weighted UniFrac distances, and diversity patterns were visualized using a principal coordinate analysis (PCoA). Taxonomic composition in RA was summarized in chord and bar plots at phylum and genus levels. Inferred functional analysis was performed using the Tax4Fun2‐based workflow implemented in the *microeco* package (v1.9.1) [46], based on the pathway reference profiles (Ref99NR) from the Kyoto Encyclopedia of Genes and Genomes (KEGG) database [47]. Similarities greater than 97% were considered orthologous KEGG groups (KOs) and “Human Diseases” pathways were removed.

### 2.8. Statistical Analysis

All statistical analyses were conducted in R (v4.5.1). Growth performance and feed utilization indicators are reported as mean ± standard deviation. After confirming normality with the Shapiro–Wilk test and homogeneity of variances using Levene’s test, differences among dietary treatments were assessed using one‐way ANOVA (*p* ≤ 0.05), followed by Tukey’s HSD post hoc test for pairwise comparisons.

Microbiota analyses were first compared across sample types (D, AI, PI, and SK), and subsequently stratified by sample type for independent analyses using the same statistical procedures described below. Differences in alpha diversity among groups were evaluated using the Kruskal–Wallis test, followed by pairwise Wilcoxon rank‐sum tests with false discovery rate (FDR) correction for multiple comparisons (*p* ≤ 0.05) [48]. Beta diversity was analyzed following confirmation of homogeneity of group dispersions via PERMDISP (*p* > 0.05) [49]. PERMANOVA [50] was then used to evaluate the effect of diet on microbial community structure. When significant overall effects were detected, pairwise PERMANOVA comparisons were performed, with FDR‐adjusted *p* values (*p* ≤ 0.05). Differentially abundant taxa among dietary groups were identified using Analysis of Compositions of Microbiomes with Bias Correction (ANCOM‐BC) [51]. Significance was defined as *p* ≤ 0.05 after FDR adjustment. Taxa with significant differences and an absolute log fold change (|LFC|) > 2 were visualized in heatmaps. In addition, a linear discriminant analysis (LDA) effect size analysis (LEfSe) was performed to test differences in the RA of inferred functions [52]. These analyses were performed using the *microeco* package, setting significance at *p* ≤ 0.05 and with a LDA threshold score >2.

Finally, Spearman’s rank correlation analysis was performed to identify potential microbial biomarkers associated with growth performance. Correlations between alpha diversity indices and the RA of differentially represented phyla and genera were evaluated against growth performance indicators. Only significant and moderate correlations (|rho| > 0.5; *p* ≤ 0.05) were retained and visualized as heatmaps.

## 3. Results

### 3.1. Growth Performance

At the end of the feeding trial, growth performance indicators differed among dietary groups (*p* ≤ 0.05) (Table 3). In particular, fish fed the AB5 diet did not differ significantly from the CTRL group in terms of SL, BW, K, WG, and SGR (*p* > 0.05). In contrast, rainbow trout fed the AB10 diet exhibited significantly lower values in SL and BW, WG, and SGR compared to both AB5 and CTRL groups (*p* ≤ 0.05). Fish fed the PO5 diet showed significantly poorer performance results in terms of SL, BW, WG, and SGR compared to fish fed the AB and CTRL diets (*p* ≤ 0.05), even though values of SL and SGR were similar to those of the AB10 group (*p* > 0.05). Finally, fish fed the PO10 diet exhibited the lowest somatic growth among all dietary groups (*p* ≤ 0.05).

**Table 3:** Growth performance and feed efficiency indicators of rainbow trout (*Oncorhynchus mykiss*) fed with the experimental diets containing *A. bisporus* (AB) and *P. ostreatus* (PO) stem meals for 60 days.

Day		CTRL	AB5	AB10	PO5	PO10	*p*‐Value
0	SL (cm)	13.19 ± 0.12	13.22 ± 0.07	13.19 ± 0.08	13.13 ± 0.11	13.10 ± 0.11	>0.05
	BW (g)	22.10 ± 0.20	22.08 ± 0.31	22.12 ± 0.34	22.00 ± 0.21	22.06 ± 0.46	>0.05
60	SL (cm)	21.79 ± 0.10^a^	21.77 ± 0.10^a^	21.29 ± 0.12^b^	20.89 ± 0.12^b^	17.94 ± 0.38^c^	<0.001
	BW (g)	172.81 ± 2.05^a^	173.57 ± 1.90^a^	160.20 ± 1.57^b^	151.65 ± 1.36^c^	92.37 ± 5.43^d^	<0.001
	WG (g)	150.70 ± 2.02^a^	151.49 ± 2.09^a^	138.08 ± 1.34^b^	129.64 ± 1.41^c^	70.31 ± 5.34^d^	<0.001
	SGR (%)	3.43 ± 0.02^a^	3.44 ± 0.04^a^	3.30 ± 0.02^b^	3.22 ± 0.02^b^	2.38 ± 0.10^c^	<0.001
	K	1.67 ± 0.04^a^	1.68 ± 0.03^a^	1.66 ± 0.03^a^	1.67 ± 0.02^a^	1.60 ± 0.02^b^	0.005
	FI (g/tank)	4681.51 ± 8.75^a^	4688.01 ± 10.56^a^	4675.22 ± 15.39^a^	4539.84 ± 22.50^b^	2807.52 ± 48.93^c^	<0.001
	FCR	0.86 ± 0.01^c^	0.85 ± 0.02^c^	0.93 ± 0.01^bc^	0.96 ± 0.01^b^	1.13 ± 0.08^a^	<0.001

*Note*: Values are expressed as mean ± SD (*n* = 4 replicate tanks per diet). Different superscript letters within the same row indicate significant differences (*p* ≤ 0.05). CTRL, Control diet; AB5, *A. bisporus* diet (50 g kg^−1^); AB10, *A. bisporus* diet (100 g kg^−1^); PO5, *P. ostreatus* diet (50 g kg^−1^); PO10, *P. ostreatus* diet (100 g kg^−1^).

Abbreviations: BW, body weight; FCR, feed conversion ratio; FI, total feed intake; K, condition factor; SGR, specific growth rate; SL, standard length; WG, weight gain.

Regarding feed utilization parameters, FI was not affected in both AB diets regardless of the level of inclusion considered (50 and 100 g kg^−1^), whereas FI values were significantly reduced in rainbow trout fed PO5 and PO10 diets (*p* ≤ 0.05). FCR was also affected by the inclusion of PO in diets for rainbow trout; in particular, similar FCR significantly increased as the levels of PO meal increased in diets compared to the CTRL group (*p* ≤ 0.05), whereas similar FCR results were found between the CTRL and AB5 and AB10 diets (*p* > 0.05).

### 3.2. Description of Microbial Communities in Experimental Feeds, Intestine, and Skin Rainbow Trout Samples

**Figure 1:**
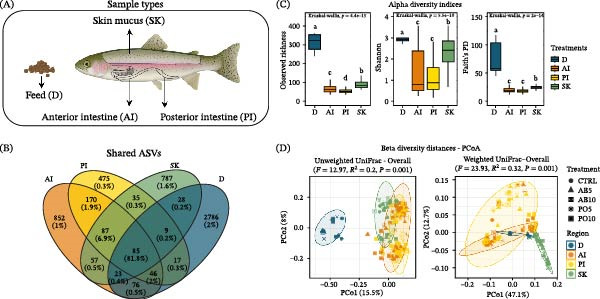
Microbiota indicators across four sample types from feed and rainbow trout (*Oncorhynchus mykiss*) fed mushroom stem‐based diets for 60 days. (A) Sample types: feed (D), anterior intestine (AI), posterior intestine (PI), and skin mucus (SK). (B) Venn diagram showing shared and unique ASVs (relative abundance). (C) Alpha diversity indices (boxplots). (D) Beta diversity (PCoA plots).

After quality control and rarefaction, a total of 4,565,440 clean reads and 5533 ASVs were obtained from 160 samples collected across the four types of samples analyzed (Figure 1A). In total, 1396, 924, 1111, and 3070 ASVs were identified in the AI, PI, SK, and D samples, respectively (Figure 1B). Among these, 85 ASVs (81.8%) were shared across all groups. AI and PI shared the highest number of ASVs (388), representing 92.6% of their total abundance. The microbiota from the SK and D samples shared more ASVs with the AI (SK–AI: 252 ASVs; 89.6%, and D–AI: 230 ASVs; 84.7%) than with the other samples. Additionally, 852, 475, 787, and 2786 ASVs were identified as unique to the AI, PI, D, and SK, respectively, with the SK samples showing the highest number of unique ASVs.

According to alpha diversity indices, the microbiota from D samples showed significantly higher values of observed richness, Shannon, and Faith’s PD indices compared to those from the SK, AI, and PI (*p* ≤ 0.05, Figure 1C). No significant differences were detected between the AI and PI for the Shannon or Faith’s PD indices (*p* > 0.05). In contrast, observed richness differed significantly among all groups, being highest in the SK, followed by the AI, and lowest in the PI samples. Regarding beta diversity, all groups exhibited distinct microbial community structures based on both Unweighted and Weighted UniFrac distances as depicted in Figure 1D (*p* ≤ 0.05).

**Figure 2:**
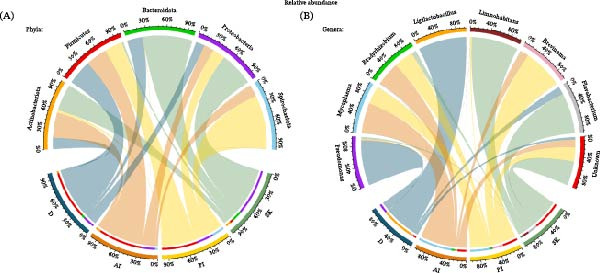
Microbiota indicators across four sample types from feed and rainbow trout (*Oncorhynchus mykiss*) fed mushroom stem‐based diets for 60 days. (A) Relative abundance of dominant phyla in each microbiota type of sample and (B) Dominant genera in each microbiota sample.

Regarding data on RA, the most dominant phyla in D, AI, and PI samples were Firmicutes (RA = 73% in the D, 64% in the AI, 53% in the PI) and Proteobacteria (RA = 18% in the D, 20% in the AI, 26% in the PI), whereas in the SK the most dominant phyla were Proteobacteria (69%), Bacteroidota (17%), and Actinobacteriota (8%) (Figure 2A). Spirochaetota was one of the most abundant phyla in the PI (19%), but it was present in smaller proportions in the AI (4%). At genus level, the dominant genera in the D were *Ligilactobacillus* (43%) and *Pseudomonas* (12%), whereas in the intestine were *Mycoplasma* (AI = 54% and PI = 33%), *Brevinema* (AI = 4% and PI = 19%) and *Bradyrhizobium* (AI = 6% and PI = 5%); and in the SK were *Flavobacterium* (16%) and *Limnohabitans* (13%) (Figure 2B). Once the differences among host‐associated matrices were characterized, the following sections describe the dietary effects on each sample type.

### 3.3. Feed Microbiota

**Figure 3:**
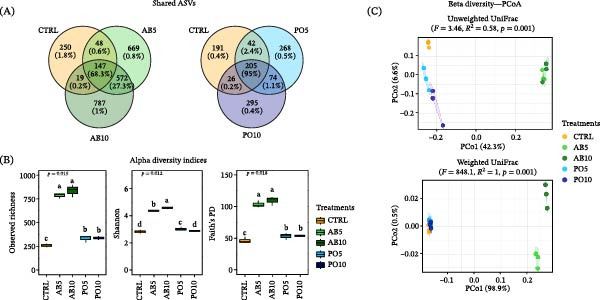
Microbiota indicators used to compare microbial communities in the experimental feeds (D) for rainbow trout (*Oncorhynchus mykiss*) containing different levels of mushroom stem‐meals of *Agaricus bisporus* (AB5, 50 g kg^−1^ and AB10, 100 g kg^−1^) and *Pleurotus ostreatus* (PO5, 50 g kg^−1^ and PO10, 100 g kg^−1^). (A) Venn diagram of shared ASVs between treatments and CTRL groups. (B) Alpha diversity indices (boxplots). (C) Beta diversity (PCoA plots).

The microbiota of experimental feeds revealed that AB5 and AB10 diets shared 68.3% of their RA with the CTRL diet (147 ASVs), whereas PO5 and PO10 diets shared 95% with the CTRL (205 ASVs) (Figure 3A). Accordingly, alpha diversity indices revealed significant differences among experimental feeds for all diversity metrics (*p* ≤ 0.05) (Figure 3B). Regarding beta diversity, although PERMANOVA detected overall differences among experimental diets based on Unweighted (*F* = 3.46, *R* ^2^ = 0.58, *p* = 0.001) and Weighted Unifrac distance (*F* = 848.10, *R* ^2^ = 1.00, *p* = 0.001), FDR‐adjusted pairwise comparisons revealed no significant differences between groups that might be attributed to the low number of replicates per feed analyzed (Figure 3C).

**Figure 4:**
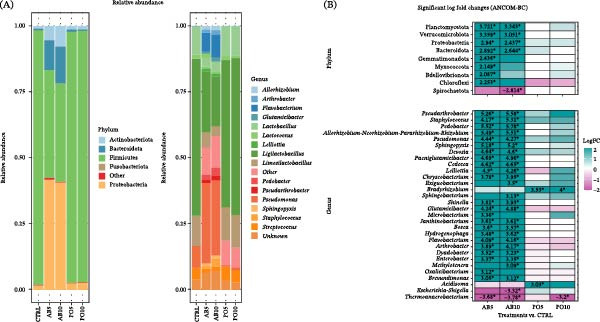
Microbiota indicators used to compare microbial communities in the experimental feeds (D) for rainbow trout (*Oncorhynchus mykiss*) containing different levels of mushroom stem‐meals of *Agaricus bisporus* (AB5, 50 g kg^−1^ and AB10, 100 g kg^−1^) and *Pleurotus ostreatus* (PO5, 50 g kg^−1^ and PO10, 100 g kg^−1^). (A) Bar plots showing the relative abundance of dominant phyla and genera (prevalence > 15% and RA > 1%). “Other” includes taxa that did not meet these parameters. (B) Heatmap showing significant differential abundance (log fold change, |LFC| > 2; *p* ≤ 0.05) for each inclusion level and mushroom‐based feed compared with the CTRL feed at the phylum and genus level.

According to the compositional abundance of feeds, the dominant phyla and genera are shown in Figure 4A. Significant differential abundances (*p* ≤ 0.05; |LFC| > 2) were detected in nine phyla, while eight phyla were more abundant in the AB5 diet (including the dominant phyla Proteobacteria and Bacteroidota) and three in the AB10 compared to the CTRL diet, and only one phylum (Spirochaetota, a non‐dominant taxon) was less abundant in the AB10 feed (Figure 4B). No significant differences at phylum‐level composition were observed between the PO5 and PO10 and the CTRL diet (*p* > 0.05). At the genus level, 30 genera with significant differences (*p* ≤ 0.05; |LFC| > 2) relative to the CTRL diet.

### 3.4. AI Microbiota

**Figure 5:**
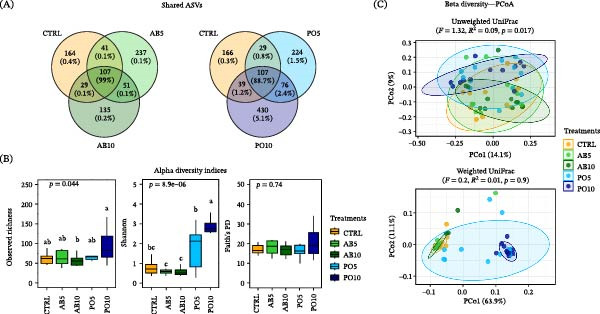
Microbiota indicators used to compare microbial communities in the anterior intestine (AI) of rainbow trout (*Oncorhynchus mykiss*) fed mushroom stem‐based diets containing two inclusion levels of *Agaricus bisporus* (AB5, 50 g kg^−1^ and AB10, 100 g kg^−1^) and *Pleurotus ostreatus* (PO5, 50 g kg^−1^ and PO10, 100 g kg^−1^) for 60 days. (A) Venn diagram of shared ASVs between treatments and CTRL groups. (B) Alpha diversity indices (boxplots). (C) Beta diversity (PCoA plots).

The AI microbiota revealed that fish fed the CTRL and both AB diets shared 99% of their RA (107 ASVs), whereas those fed both PO diets shared 88.7% with the CTRL group (107 ASVs) (Figure 5A). Alpha diversity indices revealed no significant differences between the microbiota of fish fed both AB diets and the CTRL diet (*p* > 0.05). In contrast, only the PO10 diet resulted in a higher Shannon index in the AI (*p* < 0.001) compared to the rest of diets (Figure 5B), as well as significantly higher observed richness compared to the AB10 diet. The Faith’s PD index did not show significant differences among groups (*p* > 0.05). Regarding beta diversity for AI samples, PERMANOVA did not detect significant differences based on weighted UniFrac distances (*F* = 0.20, *R* ^2^ = 0.01, *p* = 0.9), but it did detect differences using unweighted UniFrac distances (*F* = 1.32, *R* ^2^ = 0.09, *p* = 0.02) (Figure 5C). In this analysis, rainbow trout specimens fed the AB5, AB10, and CTRL diets showed no significant differences among them, whereas fish fed PO5 and PO10 diets differed both between each other and when compared to fish fed the AB and CTRL diets (*p* ≤ 0.05).

**Figure 6:**
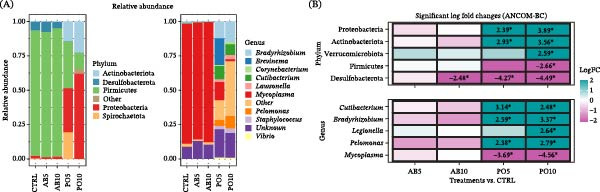
Microbiota indicators used to compare microbial communities in the anterior intestine (AI) of rainbow trout (*Oncorhynchus mykiss*) fed mushroom stem‐based diets containing two inclusion levels of *Agaricus bisporus* (AB5, 50 g kg^−1^ and AB10, 100 g kg^−1^) and *Pleurotus ostreatus* (PO5, 50 g kg^−1^ and PO10, 100 g kg^−1^) for 60 days. (A) Bar plots showing the relative abundance of dominant phyla and genera (prevalence > 25% and RA > 0.1%). “Other” includes taxa that did not meet these parameters. (B) Heatmap showing significant differential abundance (log fold change, |LFC| > 2; *p* ≤ 0.05) for each experimental diet compared with the CTRL group at the phylum and genus level.

Fish fed AB5, AB10, and the CTRL diets displayed a similar microbial composition, with dominant phyla (RA > 1%) including Firmicutes (91%−93%), Desulfobacterota (5%−7%), and Proteobacteria (1%−2%) (Figure 6A). In contrast, the microbiota of fish fed both PO diets was dominated by Proteobacteria (32%−62%), Firmicutes (12%−33%), Actinobacteriota (14%−22%), and specifically in the PO5 group, Spirochaetota (19%) and Plantomycetota (1%) in the PO10 diet. According to the ANCOM‐BC analysis, fish fed the AB5 diet showed no significant differences in phylum‐level abundance, whereas those fed AB10 exhibited a significantly lower abundance of Desulfobacterota (Figure 6B). In contrast, PO5 and PO10 diets induced level‐dependent changes (|LFC| > 2; *p* ≤ 0.05), characterized by increased abundances of Proteobacteria, Actinobacteriota, and Verrucomicrobiota and decreased abundances of Firmicutes and Desulfobacterota relative to the CTRL group.

At the genus level, *Mycoplasma* (85%−87%) predominated in fish fed the AB and CTRL diets, while a distinct distribution was observed in those fed both PO diets. In particular, PO5‐fed fish were dominated by *Mycoplasma* (13%), *Bradyrhizobium* (12%), *Cutibacterium* (10%), *Pelomonas* (4%), *Staphylococcus* (3%), and *Lawsonella* (2%). In rainbow trout fed the PO10 diet, dominant genera included *Bradyrhizobium* (15%), *Pelomonas* (9%), *Cutibacterium* (8%), *Staphylococcus* (3%), *Lawsonella* (3%), and *Mycoplasma* (1%) (Figure 6A). Only the PO diets induced significant changes compared to the CTRL group, characterized by increased abundance of *Cutibacterium*, *Bradyrhizobium*, *Legionella*, and *Pelomonas*, and a decrease in *Mycoplasma*. These effects were generally level‐dependent, except for *Cutibacterium*, which was more abundant in rainbow trout fed the PO5 diet (Figure 6B). No significant differences among groups were observed in the functional analysis in this intestinal region (*p* > 0.05).

### 3.5. PI Microbiota

**Figure 7:**
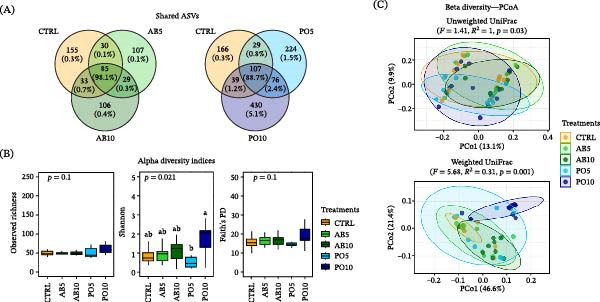
Microbiota indicators used to compare microbial communities in the posterior intestine (PI) of rainbow trout (*Oncorhynchus mykiss*) fed mushroom stem‐based diets containing two inclusion levels of *Agaricus bisporus* (AB5, 50 g kg^−1^ and AB10, 100 g kg^−1^) and *Pleurotus ostreatus* (PO5, 50 g kg^−1^ and PO10, 100 g kg^−1^) for 60 days. (A) Venn diagram of shared ASVs between treatments and CTRL groups. (B) Alpha diversity indices (boxplots). (C) Beta diversity (PCoA plots).

The analysis of microbiota of the PI revealed that fish fed the CTRL and both AB diets shared 98.1% of their RA (85 ASVs), whereas those fed the PO diets shared 88.1% with CTRL (107 ASVs) (Figure 7A). Alpha diversity indices revealed no significant differences between fish fed AB5 and AB10 diets and the CTRL diet (*p* > 0.05) (Figure 7B). Although no differences were observed between fish fed both PO feeds and the CTRL diet, the PO10‐fed fish exhibited significantly higher Shannon index compared to those from the PO5 group. Regarding beta diversity, PERMANOVA detected significant differences among groups based on unweighted UniFrac distances (*F* = 1.41, *R^2^* = 1.00, *p* = 0.03); however, FDR‐adjusted *p*‐values did not confirm these differences in the pairwise comparisons (Figure 7C). In contrast, Weighted UniFrac distances (*F* = 5.68, *R^2^* = 0.31, *p* = 0.001) revealed significant differences among all groups, except for the AB5 group, which was not significantly different from the CTRL and AB10 groups (*p* > 0.05).

**Figure 8:**
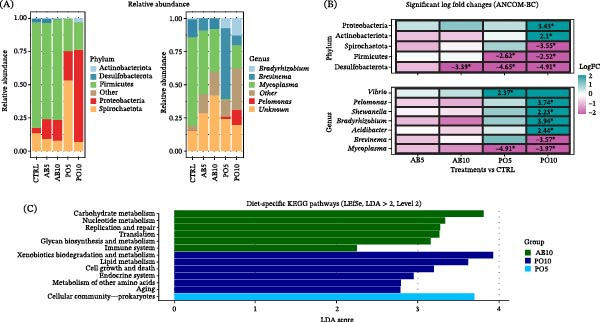
Microbiota indicators used to compare microbial communities in the posterior intestine (PI) of rainbow trout (*Oncorhynchus mykiss*) fed mushroom stem‐based diets containing two inclusion levels of *Agaricus bisporus* (AB5, 50 g kg^−1^ and AB10, 100 g kg^−1^) and *Pleurotus ostreatus* (PO5, 50 g kg^−1^ and PO10, 100 g kg^−1^) for 60 days. (A) Bar plots showing the relative abundance (RA) of dominant phyla and genera (prevalence > 25% and RA > 0.1%). “Other” includes taxa that did not meet these parameters. (B) Heatmap showing significant differential abundance (log fold change, |LFC| > 2; *p* ≤ 0.05) for each experimental diet compared with the CTRL group at the phylum and genus level. (C) LEfSe analysis of KEGG functional pathways at level 2 (LDA > 2; *p* ≤ 0.05).

Similar to the AI, fish fed both AB diets, and the CTRL diet showed a comparable microbial composition in the PI, with dominant phyla (RA > 1%) like Firmicutes (73%−79%), Proteobacteria (4%–16%), Spirochaetota (8%−13%) and Desulfobacterota (1%−3%) (Figure 8A). In contrast, the microbiota of fish fed both PO diets was dominated by Proteobacteria (22%−69%), Spirochaetota (7%−53%), Firmicutes (21%−24%), and specifically in the PO10 group, by Actinobacteriota (3%). According to the ANCOM‐BC analysis, fish fed PO diets showed several significant changes compared to the CTRL group (|LFC| > 2; *p* ≤ 0.05) (Figure 8B), while among AB‐fed fish, only those fed the AB10 diet showed a significantly lower abundance of Desulfobacterota. Rainbow trout fed the PO5 diet showed significantly lower abundances of Firmicutes and Desulfobacterota, whereas their congeners fed the PO10 diet also had a lower abundance of Spirochaetota and significantly higher levels of Proteobacteria and Actinobacteriota.

At the genus level, *Mycoplasma* (33%−67%), *Brevinema* (8%−14%), *Plesiomonas* (2%−12%), were the most abundant genera in fish fed both AB and CTRL diets, while a distinct distribution was observed in rainbow trout fed both PO diets. In particular, the PI of fish fed the PO5 diet was dominated by *Brevinema* (53%), *Bradyrhizobium* (8%) and *Pelomonas* (2%), whereas those specimens fed the PO10 diet showed *Mycoplasma* (17%), *Bradyrhizobium* (13%), *Pelomonas* (11%), and *Brevinema* (7%) as the most abundant genera (Figure 8A). Significant differential RA were identified among groups (|LFC| > 2; *p* ≤ 0.05); in particular, fish fed the PO5 diet had a significantly higher abundance of *Vibrio* and lower abundance of *Mycoplasma*, while those fed the PO10 diet exhibited significantly higher abundances of *Pelomonas*, *Shewanella*, *Bradyrhizobium*, and *Acidibacter*. Among these, *Vibrio* (RA: PO5 = 0.8%), *Shewanella* (RA: PO10 = 1.9%) and *Acidibacter* (RA: PO10 = 0.6%) were considered as rare taxa due to their lower abundance in the PI microbiota (Figure 8B).

Based on the KEGG database, functional prediction analysis revealed potential metabolic pathways associated with the intestinal microbiota of fish fed the experimental diets. LEfSe analysis identified treatment‐discriminant functional categories exclusively in the PI region, with higher inclusion levels (100 g kg^−1^) resulting in a larger number of functional shifts (Figure 8C). Particularly, rainbow trout fed the AB10 diet showed higher LDA scores in pathways at level 2 related to carbohydrate (specific pathways at level 3: ko00520, ko00500, ko00051, ko00010, ko00052, and ko00562) and nucleotide metabolism (ko00240 and ko00230), replication and repair (ko03440, ko03430, ko03030, ko03420, and ko03410), translation (ko03010 and ko00970), glycan biosynthesis and metabolism (ko00511) and immunity. In contrast, fish fed both PO diets differed from those fed the AB diets but were similar to their congeners fed the CTRL diet. Specifically, fish fed the PO10 diet showed higher LDA scores in pathways related to xenobiotics biodegradation and metabolism (ko00984, ko00365, ko00364, ko00791, ko00624, ko00625, ko00643, ko00361, ko00982, ko00980, ko00930, ko00627, and ko00362), lipid metabolism (ko00140, ko00072, ko01040, ko00061, and ko00071), cell growth and death (ko04216 and ko04113), endocrine system (ko04920 and ko03320), metabolism of other amino acids (ko00410 and ko00480), and aging (ko04213). In fish fed the PO5 diet, the most prominent functional category was cellular community‐prokaryotes.

### 3.6. SK Microbiota

**Figure 9:**
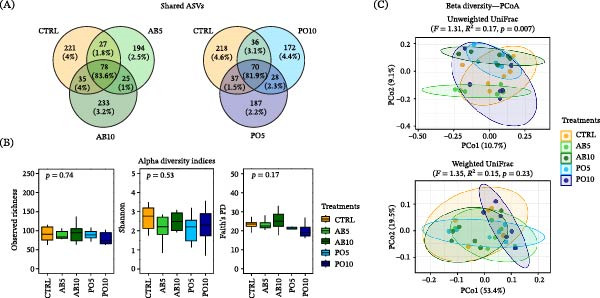
Microbiota indicators used to compare microbial communities in the skin mucus (SK) of rainbow trout (*Oncorhynchus mykiss*) fed mushroom stem‐based diets containing two inclusion levels of *Agaricus bisporus* (AB5, 50 g kg^−1^ and AB10, 100 g kg^−1^) and *Pleurotus ostreatus* (PO5, 50 g kg^−1^ and PO10, 100 g kg^−1^) for 60 days. (A) Venn diagram of shared ASVs between treatments and CTRL groups. (B) Alpha diversity indices (boxplots). (C) Beta diversity (PCoA plots).

The microbiota of SK samples from rainbow trout fed the CTRL and both AB diets shared 83.6% of their RA (78 ASVs), while those fed both PO diets shared 81.9% (70 ASVs) with the CTRL group (Figure 9A). Alpha diversity indices revealed no significant changes among experimental groups (*p* > 0.05) (Figure 9B). Regarding beta diversity, PERMANOVA did not detect significant differences based on weighted UniFrac distances (*F* = 1.35, *R* ^2^ = 0.15, *p* = 0.23), but did detect differences based on unweighted UniFrac distances (*F* = 1.31, *R* ^2^ = 0.17, *p* = 0.007). However, FDR‐adjusted *p*‐values revealed no significant pairwise differences among dietary groups (*p* > 0.05) (Figure 9C).

**Figure 10:**
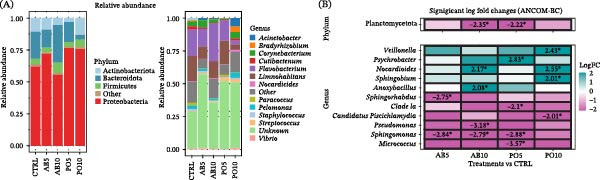
Microbiota indicators used to compare microbial communities in the skin mucus (SK) of rainbow trout (*Oncorhynchus mykiss*) fed mushroom stem‐based diets containing two inclusion levels of *Agaricus bisporus* (AB5, 50 g kg^−1^ and AB10, 100 g kg^−1^) and *Pleurotus ostreatus* (PO5, 50 g kg^−1^ and PO10, 100 g kg^−1^) for 60 days. (A) Bar plots showing the relative abundance (RA) of dominant phyla and genera (prevalence > 25% and RA > 0.5%). “Other” includes taxa that did not meet these parameters. (B) Heatmap showing significant differential abundance (log fold change, |LFC| > 2; *p* ≤ 0.05) for each experimental diet compared with the CTRL group at the phylum and genus level.

In terms of relative composition, all groups shared a similar SK microbiota profile, with the dominant phyla (RA > 1%) being Proteobacteria (56%−76%), Bacteroidota (4%−29%), Actinobacteriota (3%−13%) and Firmicutes (4%−8%) (Figure 10A). Differential RA analysis revealed a significantly lower abundance of Planctomycetota in fish fed the AB10 and PO5 diets (Figure 10B). At the genus level, dominant genera included *Flavobacterium* (3%−20%), *Limnohabitans* (4%−20%), *Acinetobacter* (1%−7%), *Corynebacterium* (1%−5%), *Pelomonas* (1%−4%), *Cutibacterium* (1%−3%), *Streptococcus* (1%−3%), *Bradyrhizobium* (0%−4%), *Paracoccus* (0%−2%), *Nocardioides* (0%−2%), *Micrococcus* (0%−1%), *Vibrio* (0%−1%), and *Staphylococcus* (0%−1%) (Figure 10A). Similar to the phylum level, no clear patterns at genus level were observed related to mushroom species or inclusion level. Most genera exhibited similar trends in abundance (either increasing or decreasing) relative to the CTRL group (Figure 10B). However, certain genera showed significantly higher abundances, such as *Nocardioides* (0.5%) and *Anoxybacillus* (5.4%) in the AB10 group, *Psychrobacter* (0.5%) in the PO5 group, and *Veillonella* (1%), *Nocardioides* (1.6%) and *Sphingobium* (0.2%) in fish fed the PO10 diet. In contrast, reduced abundances were observed for *Sphingorhabdus* (0%) and *Sphingomonas* (0.5%) in samples from the AB5 group; *Pseudomonas* (0%) and *Sphingomonas* (0.5%) in fish fed the AB10 diet; *Clade Ia* (0%), *Sphingomonas* (0%), and *Micrococcus* (0.1%) in the PO5 group; and *Candidatus Piscichlamydia* (0%) in the PO10 group. Among these, only *Nocardioides* and *Micrococcus* were part of the dominant genera in the SK microbiota of rainbow trout.

### 3.7. Potential Microbial Biomarkers Associated With Growth Performance and Feed Utilization

**Figure 11:**
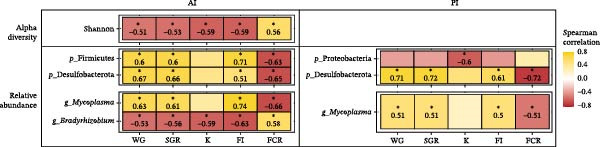
Potential microbial biomarkers associated to growth performance and feed efficiency indicators of rainbow trout (*Oncorhynchus mykiss*) fed mushroom stem‐based diets containing two inclusion levels of *Agaricus bisporus* (AB5, 50 g kg^−1^ and AB10, 100 g kg^−1^) and *Pleurotus ostreatus* (PO5, 50 g kg^−1^ and PO10, 100 g kg^−1^) for 60 days. Heatmap showing significant Spearman correlations (|rho| > 0.5, *p* ≤ 0.05) between growth performance indicators and differentially abundant taxa at phylum and genus level.

To explore potential microbial biomarkers linked to growth and feed utilization, Spearman’s rank correlations were performed between alpha diversity indices and the RA of differentially represented phyla and genera from the AI and PI samples against growth performance and feed utilization parameters. Only moderate to strong correlations (|rho| > 0.5; *p* ≤ 0.05) were considered biologically relevant (Figure 11). Regarding the AI, the Shannon diversity index showed a positive correlation with FCR and negative correlations with WG, FI, and SGR values, suggesting that higher microbial diversity was not necessarily associated with improved somatic growth performance. At the phylum level, Firmicutes and Desulfobacterota abundances were positively correlated with WG, FI, and SGR and negatively correlated with FCR, suggesting a potential beneficial role in nutrient assimilation. At the genus level, *Mycoplasma* followed the same trend as Firmicutes, showing positive associations with somatic growth indicators, whereas *Bradyrhizobium* displayed the opposite pattern, including a negative correlation with the K.

Considering the PI, alpha diversity indices showed no significant correlations with growth parameters (*p* > 0.05). However, the abundance of Proteobacteria was negatively correlated with K, while Desulfobacterota showed strong positive correlations with WG, FI, and SGR, and a negative correlation with FCR (|rho| > 0.6; *p* ≤ 0.05). A similar trend was observed for *Mycoplasma*, which exhibited moderate but significant positive correlations with the same somatic growth indicators. Although some differential taxa were identified in SK microbiota (Figure 10B), no significant correlations were detected.

## 4. Discussion

A previous study demonstrated the potential use of AB and PO stem meals as sustainable and circular ingredients for rainbow trout aquafeeds due to their nutritional quality and digestibility [29, 30]. Building on these findings, the present study evaluated the effects of replacing SBM at 25% and 50% (50 g kg^−1^ and 100 g kg^−1^ inclusion, respectively) by AB and PO stem meals on growth performance and host‐associated microbiota in rainbow trout. Overall, the AB based meals exerted slight inclusion‐dependent effects, with no impact at 50 g kg^−1^ of inclusion and moderate reductions in growth performance when included at 100 g kg^−1^ while maintaining FI and Fulton’s condition factor, whereas PO diets produced a clear level‐dependent reduction in growth and FI, particularly at 100 g kg^−1^.

In terms of growth performance, the present results differ from those previously reported by Saromines et al. [29, 30], considering that in the aforementioned study the statistical analysis included total dietary protein percentage as a covariate, since (unlike in the present experiment) the experimental diets were not isoproteic due to the high inclusion of stem meals (300 g kg^−1^) required to comply with digestibility study standards of new raw materials (diets with 245 g kg^−1^ of FM). Nevertheless, in absolute terms, the overall trend observed remains consistent with that of the present experiment. Indeed, the former authors reported that high levels of the AB meal (300 g kg^−1^) reduced CP digestibility without compromising somatic growth performance, which may explain the slight growth reduction in AB10 despite similar FI values. In addition, higher FM generally provides superior diet digestibility, buffering the effects of less digestible ingredients [53, 54]. Thus, rainbow trout fed practical/low FM diets appear to tolerate the inclusion of AB up to 50 g kg^−1^ without adverse effects in terms of growth and FI, whereas higher levels compromised growth performance due to reduced CP digestibility [29]. In contrast, diets containing AB meals have shown positive effects on other fish species fed higher FM inclusion levels. For instance, common carp (*Cyprinus carpio*) fed diets containing white button mushroom powder (5–20 g kg^−1^; FM inclusion level: 400 g kg^−1^) exhibited improved WG, SGR, and FCR, along with enhanced mucus immune activity [55]. Similarly, African catfish (*Clarias gariepinus*) exposed to *Flavobacterium columnare* and fed AB meal (10–100 g kg^−1^; FM inclusion level: 200 g kg^−1^) showed better growth performance than those fed to the control diet [56].

Conversely, PO diets impaired growth primarily through reduced FI (−3% and −40% in PO5 and PO10, respectively) since no reduction in CP digestibility was previously reported [29]. Previous studies have reported variable effects when *Pleurotus* meals were included in aquafeeds. For instance, *Pleurotus pulmonarius* stalk meal at 25–100 g kg^−1^ in FM‐free diets did not affect growth performance of African catfish [31]. In Nile tilapia (*Oreochromis niloticus*), *Pleurotus djamor* var. *roseus* based diets improved growth at moderate FM replacement levels of 20%–40% (198–265 g kg^−1^ inclusion; FM level in the control diet: 343 g kg^−1^), but performance declined at 100% FM replacement (329 g kg^−1^) [57]. Similarly, Nile tilapia tolerated up to 25% SBM replacement (110 g kg^−1^ *P. djamor*; 150 g kg^−1^ FM), whereas growth and FI decreased from 50% FM replacement (220–440 g kg^−1^) onwards [58], suggesting that diet palatability was affected at higher *Pleurotus* meal inclusion levels. Consistent with our results, FI is tightly linked to sensory acceptance in fish [59]. Multiple hypotheses, which are not mutually exclusive, may account for the reduced FI and lack of compensatory intake observed in the PO‐fed trout, including the presence of antinutritional factors (ANFs), high levels of dietary fiber, and secondary metabolites [60, 61]. In particular, PO contains ANFs such as phytic acid (424–599 mg kg^−1^) and tannins (1980–2033 mg kg^−1^), which may affect diet palatability due to their bitter and astringent properties [62], as well as impair mineral bioavailability and protein digestibility [60, 63]. In addition, dietary fiber levels above 3%–5% are not recommended for carnivorous fish [64], as they prolong gastric evacuation, reduce nutrient absorption, and limit compensatory intake, ultimately leading to growth depression [65]. Although fiber levels in this study were below that threshold, the PO5 and PO10 diets contained higher fiber contents (15 and 18 g kg^−1^, respectively) than the CTRL diet (10 g kg^−1^), which may have contributed to reduced feed utilization and growth. Another potential explanation would be the overstimulation of fish due to high levels of dietary β‐glucans. In particular, PO stem meal contained nearly five times more β‐glucans than AB meal (30.1% vs. 5.7%), with more complex β‐(1→3,1→6) linkages [66] compared to the simpler β‐(1→6) structures [67]. This higher structural complexity may enhance immune activation and elevate fish metabolic energy demand. Consequently, the reduced FI, which lowers dietary energy availability, combined with immune system overstimulation may contribute to explain the growth impairments observed at high PO meal inclusion levels [57, 58, 68]. Nevertheless, further immunological approaches are required to confirm this hypothesis.

### 4.1. The Mild Prebiotic Properties of AB Stem Meal in Rainbow Trout Diets

Although the inclusion of AB meals in aquafeeds has shown good performance results in several fish species [55, 56], its effects on fish microbiota has been less studied [30]. In higher vertebrates, AB has been shown to modulate gut microbiota; for instance, pigs fed freeze‐dried AB (equivalent to 1–2 g fresh kg^−1^ BW) exhibited no changes in somatic growth or in the alpha diversity in gut microbiota, but showed increased abundances of Lachnospiraceae and Ruminococcaceae [69]. Similarly, fermentation of AB polysaccharides by human fecal microbiota altered Shannon and Simpson diversity indices without affecting richness, promoting beneficial genera such as *Phascolarctobacterium* and *Parabacteroides* [70]. In the current study, the administration of the AB5 diet resulted in no remarkable intestinal microbiota changes, whereas the AB10 diet slightly reduced Desulfobacterota (family Desulfovibrionaceae) without affecting alpha or beta diversity indices. Desulfobacterota are anaerobic sulfate‐reducing bacteria that preferentially utilize sulfur‐rich substrates [71]. The AB meals contained lower levels of sulfur amino acids (Met + Cys: 1.19%) than the SBM in the CTRL diet (Met + Cys up to 1.85%; INRA [72], which may partially explain this reduction. Although evidence in fish is limited, Desulfovibrionaceae have shown both positive and negative associations with host condition: increased abundance following antibiotic treatment or restraint stress in common carp [73], and concomitant increases with immune gene up‐regulation in rainbow trout fed yeast‐derived components [74]. These results highlight the sensitivity of this taxon to dietary and physiological variations. In the present study, Desulfobacterota abundance was reduced by mushroom meals, whereas this reduction was positively correlated with rainbow trout somatic growth in both intestinal regions, suggesting a potentially beneficial role in the host. These contrasting results indicate that the functional contribution of Desulfobacterota likely depends on community composition and inter‐species interactions rather than abundance alone, as microbial effects often arise from community‐wide restructuring and emergent functions [75].

Despite the modest taxonomic changes observed in the PI of AB10‐fed fish, the predicted functional pathways related to carbohydrate, nucleotide, and glycan metabolism, as well as genetic information processing (replication, repair, and translation) and immune activity, were up‐regulated. Similarly, carbohydrate‐associated functional responses have been reported in Nile tilapia fed polysaccharide‐based prebiotics (0.5%–0.75% *Aloe vera* extract) [76] and European seabass (*Dicentrarchus labrax*) and gilthead seabream (*Sparus aurata*) fed diets in which 30% of FM was replaced by insect meal containing *ca*. 3% more fiber and carbohydrates [77]. These functional shifts likely reflect microbial metabolic adaptations to dietary polysaccharides rather than direct host immune activation, suggesting that AB meals may enhance beneficial microbial activity through metabolic modulation without extensive bacterial community restructuring.

### 4.2. PO Stem Meal Alters the Gut Microbial Intestinal Community in Rainbow Trout

*Pleurotus* spp. have been widely studied for their prebiotic potential and β‐glucan content [78], although most microbiota research has focused on fermented forms, which generally show only minor modulatory effects on gut microbiota [31, 33]. In contrast, the present study revealed clear level‐dependent effects of PO stem meals on the intestinal microbiota of rainbow trout. Alpha diversity indices increased in both the AI and PI (PO10 > CTRL; PO > AB), while beta diversity analyses (Weighted UniFrac) showed distinct separation of PO‐fed groups from AB and CTRL, indicating that *Pleurotus*‐based diets altered community composition in a level‐dependent manner, primarily through shifts in dominant taxa. These outcomes differed from previous findings. In largemouth bass (*Micropterus salmoides*), the inclusion of up to 20% *Pleurotus eryngii* root waste combined with SBM co‐fermented protein increased Firmicutes abundance without affecting growth or alpha diversity [33], effects that were likely attributable to the fermentation process rather than the mushroom meal itself. Similarly, in African catfish, up to 100 g kg^−1^ *P. pulmonarius* stalk meal maintained somatic growth, while increasing LAB and reducing *Escherichia coli*, *Salmonella*, and *Vibrio* counts [31]. In the present study, the PO10 diet affected both intestinal regions, whereas the PO5 diet had an impact confined to the AI, indicating clear level‐ and compartment‐specific responses.

Regarding the AI, the increase in the RA of Desulfobacterota, Actinobacteriota, and Proteobacteria was accompanied by a decrease in Firmicutes, a pattern often considered unfavorable, since Firmicutes are generally associated with host health, whereas elevated Proteobacteria levels are linked to dysbiosis and inflammation in fish [79, 80]. Genus‐level analysis provided greater resolution, revealing that both PO diets caused a five‐fold reduction and near depletion of Desulfobacterota (Desulfovibrionaceae) in both AI and PI, which was coincident with the most severe decline in somatic growth. This reduction likely reflected the lower supply of sulfur‐containing amino acids in PO diets (Met + Cys: 0.56%) compared with the SBM (1.85%). Given the positive correlation between Desulfobacterota abundance and growth, maintaining adequate dietary sulfur levels may help preserve beneficial microbial communities. Furthermore, both PO diets increased *Cutibacterium* abundance in the AI, a genus commonly detected in fish intestines as beneficial [12, 40, 81, 82]. This bacterium ferments diverse carbohydrates, producing vitamin B_12_ and short‐chain fatty acids (mainly propionate) that supply energy to enterocytes and lower intestinal pH [83–86]. Although typically considered beneficial, its three‐fold increase coincided with higher fiber levels in the diet and reduced FI and poor growth, suggesting that any positive effects from SCFA production were offset by the overall energy deficit resulting from lower feed consumption found in these groups. These conditions may have favored the proliferation of secondary consumers such as *Pelomonas*, *Legionella*, and *Bradyrhizobium*, which utilize fermentation end‐products, while disadvantaging beneficial fermenters like *Mycoplasma*, known for its low tolerance to acidic environments [87].

*Pelomonas* is a common member of fish intestinal microbiota [88, 89] and is known to persist under variable nutrient conditions [90, 91]. Its abundance tends to increase with dietary polysaccharide supplementation [89, 92], likely reflecting its role as a secondary beneficiary of carbohydrate fermentation rather than a primary degrader [90]. In addition, *Legionella*, a strictly aerobic and non‐fermentative genus, also increased in PO10‐fed fish, whereas its increase may be driven by dietary fiber and carbohydrate availability as it has been associated with high‐gluten or SBM‐based diets [93, 94]. In the current study, *Bradyrhizobium* increased in both intestinal regions, and its RA was negatively correlated with somatic growth in the AI, results that might be associated with trophic competition with beneficial taxa such as *Mycoplasma*. *Bradyrhizobium*, previously reported in rainbow trout [95], possesses the ability to degrade complex carbohydrates and aromatic compounds [96] and has been enriched in Atlantic salmon fed alginate oligosaccharides [97]. In this context, *Mycoplasma*, a facultative anaerobe that is abundant and likely mutualistic in salmonids [98–102], decreased in fish fed PO diets, likely due to both limited arginine availability (3.65% in AB vs. 1.1% in PO) and competition with metabolically versatile fermenters. Unlike *Mycoplasma*, which primarily relies on glucose fermentation and arginine catabolism, *Cutibacterium* can utilize a wider range of carbohydrates to produce SCFAs [103], providing a competitive advantage under the polysaccharide‐rich conditions of PO diets.

Interestingly, the Shannon diversity index in the AI was negatively correlated with growth performance. Although greater diversity is not inherently detrimental and is often associated with gut health [104, 105], these results suggest that maintaining a functionally stable and balanced community, rather than elevated diversity per se, may better support fish performance [106]. This negative association likely reflected an increased evenness driven by the proliferation of low‐abundance taxa accompanied by a decline in dominant. This interpretation is supported by the lower FI observed in PO‐fed fish, as reduced nutrient availability in the gut can intensify microbial competition and favor the expansion of rare low‐abundance taxa. Such conditions may promote community restructuring rather than functional optimization, representing a compensatory microbial response to limited energy resources and the availability of complex or less digestible substrates [3, 107].

A similar pattern to that observed in the AI was detected in the PI, characterized by an increase in Proteobacteria and Acidobacteriota and a reduction in Firmicutes and Spirochaetota. At the genus level, changes were more pronounced; in particular, *Pelomonas*, *Bradyrhizobium*, and *Mycoplasma* showed similar trends, whereas *Shewanella*, *Acidibacter*, and *Vibrio* increased, and *Brevinema* decreased. In this sense, *Shewanella* is a facultative anaerobe capable of lipid degradation and multiple electron‐acceptor respiration [108–110], which presence has already been reported in salmonids [111, 112]. Its enrichment, particularly in PO10‐fed fish, likely reflects favorable conditions created by high polysaccharide availability and the reduction of fermenters such as *Mycoplasma* and *Brevinema*. This metabolic versatility of *Shewanella* may have provided a competitive advantage over other bacterial groups, suggesting trophic competition and aligning with the activation of lipid metabolism pathways observed in the PI as KEGG pathways pointed out. *Brevinema*, a strict anaerobic fermenter commonly dominant in the PI and linked to fish health [30, 97, 113–115], declined in rainbow trout fed the PO10 diet, findings that were consistent with substrate competition from metabolically versatile taxa such as *Shewanella*. Low‐abundance but potentially detrimental genera also increased, including *Acidibacter*, an aerobic, acidophilic bacterium rarely reported in fish [12], and *Vibrio*, which comprises both commensal and opportunistic species associated with vibriosis [116–118]. These genera may exploit ecological niches emerging after microbial dysbiosis. Notably, reduced *Mycoplasma* abundance has been associated with higher *Vibrio* susceptibility in rainbow trout [99], whereas an inverse relationship was observed in Chinook salmon (*Oncorhynchus tshawytscha*) [119], supporting a protective role of *Mycoplasma* against opportunistic infections.

From a functional point of view, the PI of fish fed the PO10 diet exhibited enrichment of KEGG pathways related to lipid, amino acid, and xenobiotic metabolism. The activation of lipid metabolism pathways is consistent with the higher abundance of *Shewanella*, while the enrichment of xenobiotic biodegradation pathways aligns with the proliferation of *Shewanella*, *Pelomonas*, *Bradyrhizobium*, and *Acidibacter*, genera known to degrade complex substrates [110, 120–122]. Such increment in the RA of the above‐mentioned taxa may also reflect the presence of ANFs in the PO meal [63], which are known to impair metabolism, digestibility, and host’s growth [123]. Finally, the enrichment of endocrine‐related pathways likely reflects stress‐induced disruptions in appetite and growth regulation, consistent with the reduced FI and poor performance observed in rainbow trout fed PO diets [124].

Altogether, current results evidenced that the PO meal markedly reshaped the microbiota in both the AI and PI. Fermentative and metabolically versatile genera (e.g., *Cutibacterium*, *Shewanella*) and secondary consumers of fermentation products proliferated, whereas less versatile core of fermenters (*Mycoplasma*, *Brevinema*) declined, suggesting trophic competition and functional reorganization within the rainbow trout gut community. The concomitant enrichment of lipid and xenobiotic metabolism pathways, together with the rise of opportunistic taxa (e.g., *Vibrio*) and reduced growth performance, indicated that PO diets, particularly at 100 g kg^−1^ inclusion, destabilized intestinal bacterial homeostasis.

### 4.3. Reduction of Skin Opportunistic Pathogens in Rainbow Trout fed Mushroom Stem Meals

The SK represents a primary defense barrier that contributes to colonization resistance, competitive exclusion, and antimicrobial compound production [8, 14, 15]. Although the effects of mushroom by‐products on skin‐associated microbiota have not been directly examined, evidence from other functional ingredients supports the existence of gut–skin axis interactions [7]. For instance, dietary supplementation with *P. eryngii* powder (≤2%) enhanced humoral innate immunity and increased the bactericidal activity of SK in koi carp (*Cyprinus carpio koi*) [19], while galactooligosaccharides modulated the expression of immune‐related genes in the SK of common carp [20].

In the present study, alpha‐ and beta‐diversity indices of the bacterial communities inhabiting the SK were unaffected by diet, yet several taxa shifted relative to the CTRL group. Genera that increased included *Veillonella*, *Psychrobacter*, *Nocardioides*, *Sphingobium*, and *Anoxybacillus*, whereas *Sphingorhabdus*, *Clade Ia*, *Candidatus Piscichlamydia*, *Pseudomonas*, *Sphingomonas*, and *Micrococcus* decreased. All these genera belong to phyla commonly inhabiting rainbow trout SK [37, 125, 126]. Among them, *Nocardioides*, one of the dominant taxa, is capable of degrading contaminants, pesticides, and aromatic compounds [127]; thus, its increase may be attributed to the content of aromatic and phenolic constituents of mushroom meals [128]. Furthermore, *Sphingobium*, which also increased, shares metabolic traits comparable to those of *Sphingomonas*, *Pseudomonas*, and *Micrococcus* [127], which decreased, suggesting that taxa with similar ecological functions may have undergone niche displacement. These shifts may result from changes in mucus metabolite profiles mediated by dietary modulation [20] and, to a lesser extent, by diet‐derived substrates present in the water leached from feed pellets [129].

Under current experimental conditions, reductions in *Pseudomonas* and *Candidatus Piscichlamydia* may be considered as particularly beneficial, as the former taxa includes opportunistic salmonid pathogens [130], and the latter has been associated with epitheliocystis outbreaks causing gill pathology [131, 132]. In parallel, the enrichment of *Anoxybacillus*, a facultative, fermentative genus ([133]; and *Veillonella*, which converts lactate into propionate and acetate [134, 135], suggests possible benefits mediated by SCFAs that may improve skin immune function [136]. Overall, dietary mushroom stem meals caused modest but meaningful shifts in the SK microbiota, enriching beneficial and adaptable taxa while suppressing opportunists, which may contribute to improved skin health through the gut–skin axis.

## 5. Conclusion

This study demonstrates that mushroom stem meals exert species‐ and level‐specific effects on rainbow trout growth and intestinal microbiota. Diets replacing SBM with AB meals produced minor impacts in rainbow trout, negligible at 50 g kg^−1^ and moderate at 100 g kg^−1^, whereas PO diets resulted in clear, level‐dependent impairments in growth and altered the gut microbial intestinal community, particularly when SBM was replaced at 100 g kg^−1^ inclusion level. Microbiota shifts were primarily driven by host–diet interactions rather than direct microbial transfer from the feed, reflecting host‐specific responses to ingredient composition and bioactive compounds. While AB diets did not significantly affect intestinal alpha or beta diversity indices, both PO diets increased alpha diversity and induced distinct community structures compared with both AB and CTRL groups. These divergent responses are linked to the higher fiber and β‐glucan content of the PO meal, which promoted the proliferation of fermentative genera (e.g., *Cutibacterium* and *Shewanella*), secondary consumers (*Pelomonas*, *Bradyrhizobium*, and *Legionella*), and opportunistic bacteria like those from the *Vibrio* genus, while reducing beneficial fermenters such as *Mycoplasma* and *Brevinema*. Such trophic competition likely disrupted core commensals and functional stability in the gut microbiome of rainbow trout, as evidenced by the enrichment of lipid and xenobiotic metabolism KEGG pathways.

In contrast, SK microbiota diversity remained stable across diets, though all mushroom‐based diets enriched metabolically versatile and potentially beneficial genera (e.g., *Anoxybacillus*, *Veillonella*) while reducing opportunists (*Pseudomonas*, *Candidatus Piscichlamydia*). Overall, these results indicated that the AB meal was more compatible with rainbow trout performance and gut microbial communities, whereas high PO inclusion altered intestinal bacterial homeostasis. Future research should validate the prebiotic potential of AB at 50 g kg^−1^ and assess lower PO inclusion levels or fermentative preprocessing to enhance digestibility and mitigate antinutritional effects.

**Nomenclature**
AB: *Agaricus bisporus*
AI: Anterior Intestine
ANCOM‐BC: Microbiome differential abundance and correlation analyses with bias correction
ANF: Antinutritional factors
ASV: Amplicon Sequence Variants
CTRL: Control
CF: Crude fiber
CL: Crude lipid
CP: Crude protein
DM: Dry matter
FDR: False Discovery Rate
FM: Fishmeal
GE: Gross energy
LAB: Lactic Acid Bacteria
LEfSe: Linear Discriminant Analysis Effect Size
LFC: Log fold change
PCoA: Principal Coordinate Analysis
PD: Phylogenetic Diversity index
PI: Posterior Intestine
PO: *Pleurotus ostreatus*
RA: Relative abundance
SBM: Soybean meal
SCFA: Short‐Chain Fatty Acids
SK: Skin mucus.

## Author Contributions

**Samira Reinoso**: data curation, formal analysis, validation, visualization, writing – original draft, writing – review & editing. **Carl John Saromines**: data curation, investigation, writing – review & editing. **Silvia Torrecillas**: investigation, methodology, supervision, validation, writing – review & editing. **Maria Luisa Tello Martín**: investigation, methodology, resources, writing – review & editing. **Margarita Pérez Clavijo**: investigation, methodology, resources, writing – review & editing. **Enric Gisbert**: conceptualization, data curation, formal analysis, funding acquisition, investigation, methodology, project administration, resources, supervision, validation, visualization, writing – review & editing.

## Funding

The authors declare that financial support was received for the research and/or publication of this article. This work was conducted within the GreenBlueCircle Project (TED2021‐132054B‐C21/C22) funded by the Ministerio de Ciencia e Innovación (Spain). Carl John Saromines was supported by a predoctoral grant from IRTA. Silvia Torrecillas is financed by a Ramón y Cajal fellowship (RYC2021‐031414‐I) funded by MICIU/AEI/10.13039/501100011033 and, as appropriate, by “ESF Investing in your future”, by “ESF+” or by “European Union NextGenerationEU/PRTR”.

## Ethics Statement

The study complied with the EU2010/63 guidelines (Guiding Principles for Biomedical Research Involving Animals) and Spanish laws (32/2007 and RD 1201/2015) and was approved by the Ethical Committee of the Institute of Agrifood Research and Technology (IRTA, Spain) for the use of laboratory animals (E‐10/2020) and the Generalitat de Catalunya (CEEA 219/2020).

## Conflicts of Interest

The authors declare no conflicts of interest.

## Data Availability

Raw sequencing data are available in The European Nucleotide Archive (ENA) under Project Accession Number PRJEB102050.
